# Self-Determination Competencies, (Dis)Agreement in Decision-Making, and Personal Well-Being of Adults with Mild Intellectual Disabilities in Hong Kong

**DOI:** 10.3390/ijerph182010721

**Published:** 2021-10-13

**Authors:** Phyllis King Shui Wong, Amy Yin Man Chow

**Affiliations:** 1 Department of Social Work, Chinese University of Hong Kong, Hong Kong, China; 2 Department of Social Work and Social Administration, University of Hong Kong, Hong Kong, China; chowamy@hku.hk

**Keywords:** personal goals, (dis)agreement in decision-making, self-determination, personal well-being, Hong Kong adults with ID

## Abstract

Background: The self-determination of people with an intellectual disability (ID) in the contexts of adulthood and Chinese culture is under-examined in the field of ID, even though the concept of self-determination has vigorously developed in recent decades. This study examined the relationship between self-determination competencies and the personal well-being of adults with mild ID in Hong Kong, as well as their personal goals and decision-making (dis)agreements with their significant others. Methods: We interviewed 170 participants using the AIR Self-Determination Scale—Chinese Version (AIR SDS-C) and the Personal Well-Being—Intellectual Disability (Cantonese) (PWI-C), along with a self-constructed questionnaire. Results: When the demographic characteristics were controlled, self-determination competencies correlated positively with personal well-being (r = 0.313, *p* < 0.001), diverse personal goals were identified, and agreement with significant others was dominant in both daily and major decision-making. Conclusions: A positive correlation between self-determination and personal well-being was confirmed in a Chinese population with mild ID. These findings expand the understanding of the types of personal goals and agreement patterns of people with mild ID and yield implications for further research and practices.

## 1. Introduction

In the past two decades, the construct of self-determination has undergone remarkable development in the field of international intellectual disability (ID), particularly for use in model building and instruction for skills enhancement in special education, evaluation, and environmental interventions [1,2]. Scholars state that self-determination is a multifaceted construct [3]. In the context of special education and disability, the perspectives of self-determination were developed predominantly as personal competencies and ecological constructs. Deci and Ryan [4] suggested that self-determination was the capacity to choose, whereas Mithaug [5] later defined self-determination as a set of capacities in addition to the capacity to choose. Self-Determination competency refers to a person’s capacity to enact the process of setting goals and attaining them based on his/her preferences and strengths. The person tries to achieve a goal by means of a plan, and can then evaluate his/her own performance in implementing the plan. When the person finds the original plan to be unsuccessful, he/she can attempt to regulate it. A self-determined person possesses a high level of competency in self-determination [6].

A social-ecological perspective proposes that the microsystem and mesosystem, such as family factors and the service environment, play important roles in influencing a person’s self-determination [7,8]. Cultural factors are also recognized as a crucial consideration in a person’s development of self-determination and the opportunities for applying it in practice [7,9]. Earlier studies conducted in North America found that opportunities to facilitate the self-determination of people with ID were more favourable in small congregate settings than in large congregate settings (e.g., living independently vs. living with family; small group homes vs. large institutions; congregate training/work settings vs. community-based training/work settings), e.g., [10,11,12,13]. One study found that the choice opportunities of adults with ID in Israel who lived with their family were greater than those who lived in group homes [14]. The results of a study in Italy replicated the finding that people with ID experienced more choice opportunities in community living contexts than in restrictive living contexts [15]. In a cross-context comparison, Spanish youths with ID enjoyed more self-determination opportunities at school than at home [16] and many of their parents had little knowledge of self-determination and exhibited an over-protective manner towards their children [17]. A recent study also observed a mediating role of environmental opportunities in the relationship between self-determination and ID level [18].

### 1.1. Personal Goal Setting by Adults with Intellectual Disabilities

Goal setting means one’s selection of goals [19] to pursue, and that one pursues what one wishes to [20]. Researchers have tried to distinguish between different types of goals. In educational contexts, these types include goals of learning (e.g., learning new things), goals of achievement (e.g., demonstrating competencies), interpersonal goals (e.g., making and keeping friendships), goals of social welfare (e.g., contributing to society), social solidarity goals (e.g., bringing honour to one’s family), and goals of social approval (e.g., gaining approval from teachers or peers) [21]. This study adopted Schalock and Verdugo’s quality of life (QOL) framework [22], which provides the comprehensive domains of life which are useful for categorizing and understanding the types of personal goals of adults with ID who no longer seek education-related goals but instead have diverse personal goals across various life domains. The QOL framework has eight dimensions: emotional well-being, material well-being, interpersonal relations, personal development, physical well-being, social inclusion, self-determination, and rights [22].

### 1.2. The Relationship between Self-Determination Competencies and Personal Well-Being

This study adopts Mithaug’s [5] definition of self-determination competencies, defining them as a person’s capacity regarding the process of setting goals and then attaining them based on his/her preferences and strengths. Self-Determination contributes to personal well-being, which is defined as the subjective dimensions of quality of life [23]. The literature shows that exercising self-determination can enhance the well-being of people with ID by providing, for instance, a higher life satisfaction and greater emotional well-being [24,25,26,27,28]. However, this relationship has not yet been tested in a Chinese population with intellectual disabilities.

### 1.3. (Dis)Agreement in Different Contexts Related to Major/Daily Decisions

Decision-Making inevitably involves other people. Myers [29] asserted that parents from individualistic cultures raise their children to be independent and able to make their own choices but that parents from collective cultures teach their children to be interdependent and mutually reliant. Bao and Lam [30] found that Chinese children were largely unconcerned about freedom of choice when choices were made by people they trusted and cared about, but that freedom of choice did matter to them when relatedness was low. Meanwhile, Wong [31] found that adults with mild ID in Hong Kong were sometimes willing to surrender decision-making power to their significant others, but that others expected their significant others to understand their unexpressed choices and respect their decisions [31].

Adults with ID encounter diverse decisions to be made, from daily situations to major events, across different stages of adult life. A recent study in Hong Kong revealed that adults with mild intellectual disabilities could make their own decisions in daily activities, such as going out for leisure or joining a centre for activities. However, they encountered less autonomy in major decisions in relation to financial matters, career choice, living arrangements, and romance [32]. This study used a quantitative method to further explore whether adults with ID agree or disagree with the opinions given by their significant others in different contexts.

### 1.4. Aims and Hypotheses

The majority of research in relation to the self-determination of people with disability was carried out in Western countries [31] and conducted in school-based contexts, whereas very little was studied in adult community environments [31,33]. In recent years, there was a call for more research which addresses one’s personal culture and promotes the person living a self-determined life [33]. In this light, the objectives of this study were to explore goal setting among Hong Kong adults with mild intellectual disabilities, the relationship between their self-determination competencies and personal well-being, and the (dis)agreements between them and their significant others when making decisions about different matters in their lives. Our findings enhance our understanding of those areas, filling in gaps in the knowledge in the contexts of adults with ID and in Chinese culture.

This study has three hypotheses: (1) the relationship between the self-determination competencies and personal well-being of Chinese adults with ID is positively correlated; (2) the personal goals of adults with ID are diverse across various life domains; and (3) the frequency of disagreements between adults with ID and their significant others would be higher when making major decisions.

## 2. Materials and Methods

### 2.1. Participants

Participants were a convenience sample of 176 adults with mild ID. Inclusion criteria were that the individual was identified as having mild ID, was aged 18 or above, was able to master basic comprehension and verbal communication skills, and expressed willingness to participate in the study. The latest official policy paper on rehabilitation in Hong Kong, the Rehabilitation Programme Plan, still uses the definition of ID from DSM-IV. It specifies four levels of severity of ID, with the mild grade denoting someone with an IQ level between 50–55 and approximately 70 and with limitations in at least two of the 10 areas of adaptive skills [34]. This study identified potential participants on the basis of the assessment results shown in the individuals’ latest psychological reports. One participant was removed due to being under 16, two participants only partially completed the questionnaire, and three others were removed because the grade of their ID was moderate rather than mild. Thus, our final analyses were based on data collected from 170 participants. The participants were 82 (48.2%) men and 88 (51.8%) women, the mean age of the group was 39.5 years (SD = 11.3), and the individuals ranged in age from 18 to 65 years old. In all, 125 (73.5%) participants had no other type of disability, whereas 44 (25.9%) participants indicated that they had other types of disability. The demographic characteristics of the participants are reported in detail in Table 1.

### 2.2. Measures

The questionnaire had five sections. The first section was an open-ended item that collected the participants’ personal goals. The second section measured the participants’ self-determination competencies (the independent variable) by using the first section of the AIR Self-Determination Scale—Chinese Version (AIR SDS-C) [35]. The third section measured personal well-being (the dependent variable) by using the Personal Well-Being—Intellectual Disability (Cantonese) 3rd Edition (PWI-C) [36]. The fourth section gathered information to investigate the patterns of contextual self-determination in situations of agreement and disagreement with significant others. The last section collected demographic data.

#### 2.2.1. Personal Goals

To collect the participants’ personal goals, we asked them the open-ended question ‘Please tell a goal you are working on’, and we allowed them to provide more than one goal. The exact wording of their answers was recorded for analysis.

#### 2.2.2. The Self-Determination Competencies Section of the AIR Self-Determination Scale—Chinese Version (AIR SDS-C)

The AIR SDS-C is the validated Chinese version of the AIR Self-Determination Scale originally developed by Wolman and colleagues [6]. It is a twenty-four-item, five-point Likert scale consisting of scores from four domains: self-determination competencies, feelings, school or workplace opportunities, and home or hostel opportunities. We conducted a confirmatory factor analysis (CFA) which revealed a very similar factor structure to the original English version. All of the factor loadings were between 0.42 and 0.76, so all items were retained. Results of the CFA suggested a relatively good fit of the data to the model (χ^2^(*df* = 246, *n* = 356) = 391.64, χ^2^/*df* = 1.59, RMSEA = 0.041 90% CI [0.033–0.048], CFI = 0.933, TLI = 0.925, SRMR = 0.05). The AIR SDS-C also showed good levels of internal reliability (α = 0.88) [35].

This study used only the self-determination competencies section of the overall scale because only the variable of self-determination competencies was an issue. This section contained six items: (1) I know what I need, what I like, and what I am good at; (2) I set goals to get what I want or need. I think about what I am good at when I do this; (3) I figure out how to meet my goals. I make plans and decide what I should do; (4) I begin working on my plans to meet goals as soon as possible; (5) I check how I am doing when I am working on my plan. If I need to, I ask others what they think of how I am doing; and (6) If my plan does not work, I try another one to meet my goals. The participants were asked to indicate how often they tended to perform the steps in their daily lives (1 = never, 2 = almost never, 3 = sometimes, 4 = almost always, and 5 = always). The reliability coefficient of this part of the study was good (α = 0.79).

#### 2.2.3. Personal Well-Being—Intellectual Disability (Cantonese) 3rd Edition (PWI-C)

The PWI-C is the Cantonese version of the Personal Well-Being Index and was validated by Cummins and Lau [36]. It is an eight-item, five-point Likert-scale questionnaire used to measure the subjective well-being of people with ID. The first item is ‘Are you happy with life as a whole?’, and that question is followed by seven items asking about how satisfied the person is with the seven QOL domains: standard of living, personal health, achievement in life, personal relationships, personal safety, community-connectedness, and future security. The Cronbach α coefficients were 0.80 and the item-total correlations ranged between 0.33 and 0.69. The Kaiser–Meyer–Olkin (KMO) values exceeded 0.80. Bartlett’s test also reached statistical significance (*p* < 0.05). The total variance explained by the domains was 48.3 [37]. The reliability coefficient of this study was also good (α = 0.71).

#### 2.2.4. Decision-Making Patterns in (Dis)Agreement Situations

Participants were asked how much agreement their usually was between their views and their significant other’s views in nine different contexts, from the contexts of decision-making in daily situations (e.g., choices about activities and the use of money) to the contexts of decision-making about life events (e.g., choices about one’s job and marriage partner) and, when there was disagreement between them and their significant others, whose viewpoint they tended to follow (Table 2). The nine contexts of different levels of autonomy were developed from a previous qualitative study conducted by Wong [32] which suggested that people with ID have a continuum of autonomy in decision-making (from more autonomous to less autonomous and from minor contextual situations to major ones).

The face validity and content validity of this section were reviewed by an expert panel consisting of three social workers and one support worker, all of whom were experienced in the field of ID. The two social workers had over 20 years of working experience in the ID setting; one was more experienced in residential settings and the other in community-support settings. The third social worker had 3 years of experience in employment services. The support worker was responsible for providing direct services and had more than 10 years of experience in day-training settings. These experts were invited to comment on the relevance of the items which reflected the daily-life situations of the adults with mild ID and on the appropriateness of the questions that the participants were expected to answer. In the panel meeting, the experts went through the questionnaire item by item and their qualitative feedback was shared. Content validity was thus ensured and the consensus on the item content was achieved through the review process.

#### 2.2.5. Demographic Information

The final section collected demographic information. Nine items of information were sought: gender, age, IQ or ID grade, presence of any other disabilities, marital status, living arrangements, educational level, monthly income, and services received.

#### 2.2.6. Pilot Test

**Figure 1:**
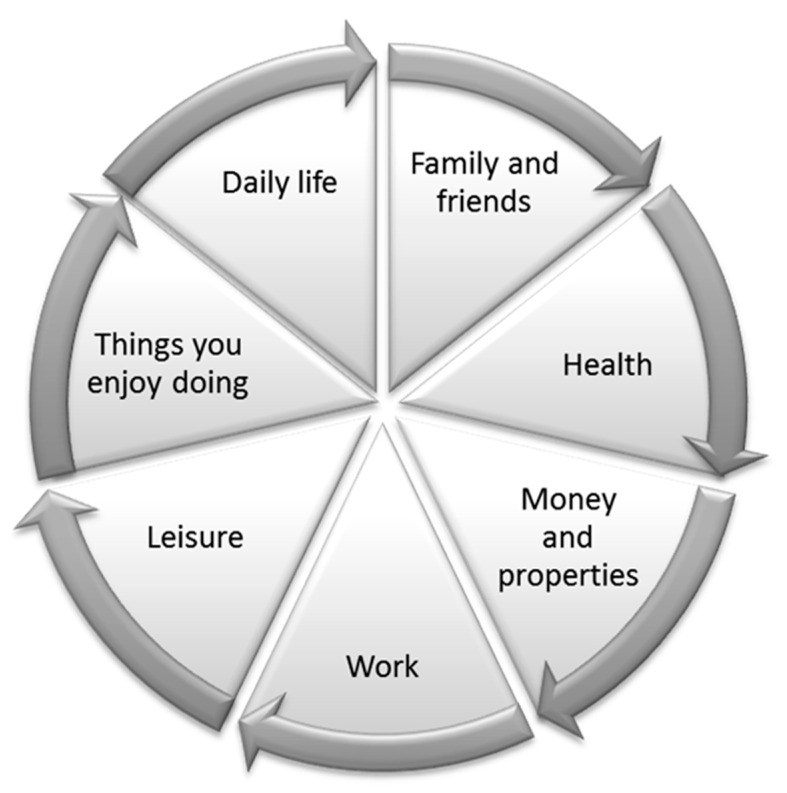
Pie Chart of ‘Life Overall’.

First, a pilot test was conducted to confirm the feasibility of the instruments. Six adults (three men and three women) with mild ID were invited to take the pilot test. Three came from sheltered workshops-cum-hostels, and the others were living in the community and using services from district support centres. The heterogeneity of the participants in terms of their gender, service settings, and living arrangements assured the questionnaire’s applicability to participants with different backgrounds. The first author and a research assistant conducted the interviews for the pilot test, with one participant at a time. During the interviews, we observed the participants’ responses and reactions and made notes immediately after the test. We also collected verbal feedback from the participants. The results showed that the participants had no problem in understanding the meanings of all six items of the second section (regarding self-determination competencies), other than Participant A, who appeared to not fully understand the meaning of frequency (i.e., never—almost never—sometimes—almost always—always). Therefore, we explained to him that he could think of ‘never’ as none, ‘almost never’ as once or twice out of 10, ‘sometimes’ as 5 times out of 10, ‘almost always’ as 7 or 8 times out of 10, and ‘always’ as 10 times out of 10. With this explanation he was able to answer the items. For the third section (the Personal Well-Being Index), three participants said that they did not understand the words ‘life overall’ in item 1. Therefore, we explained that ‘life overall’ included their family, their job, the things they were interested in doing, and so on. We then designed a visual cue ‘Pie chart of life overall’ (Figure 1) to illustrate ‘life as a whole’ for them. With that assistance, they were able to answer the questions without difficulty. For the fourth section (regarding decision-making and (dis)agreement situations), the results showed that the participants understood what the questions were asking, because they were able to give different answers instead of choosing a single option for all nine situations. The pilot test results demonstrated that the questionnaire was feasible and suggested a few effective techniques and tools.

### 2.3. Procedures

Ethical approval for the study was granted by the Human Research Ethics Committee for Non-Clinical Faculties at the University of [REDACTED] (Reference No. [REDACTED]). Formal invitation letters were sent to the headquarters of NGOs, and emails were sent to all of the adult service units which served people with mild ID in Hong Kong. The service units included employment services and residential services. The NGOs were invited to return the reply slip if they had eligible service users who showed an interest in participating, and our research assistant then followed up with arrangements for data collection.

We used a written consent form that was developed with reference to the standard format provided by the Ethics Committee. We also adopted two additional measures suggested by the International Handbook of Applied Research in Intellectual Disabilities [38] to optimize the participants’ understanding of the information before they were asked for their informed consent, as follows:

(1) Consent sessions before the questionnaire interviews were arranged by research assistants experienced in communicating with people with ID. The communicators explained the objectives and procedures of the study, the voluntary nature of the participation, and the participants’ right to withdraw at any time, using plain language and incorporating pictures as visual aids. The participants were then asked to sign the consent form.
(2) A passive consent form was also used in order to provide dual protection to the participants’ rights. Once participants had signed the individuals’ consent form, they were then also asked to give consent for their parents or guardians to be contacted for a passive written consent.

This study adopted a self-rating method via individual face-to-face interviews at participants’ service units. No proxy response was allowed. Four interviewers that were experienced in communicating with people with ID were recruited and given a three-hour training session on skills and procedures for interviewing.

### 2.4. Data Analyses

Schalock and Verdugo’s QOL framework [22] was adopted to categorize the participants’ personal goals. The framework comprised eight QOL domains: emotional well-being, material well-being, physical well-being, interpersonal relations, personal development, social inclusion, self-determination, and rights, and their respective subdomains.

The IBM SPSS Statistics software programme was used for the descriptive and inferential statistical analyses. Descriptive analysis was used for the demographic data, and the quantitative variables were described in terms of means and standard deviations. Pearson’s correlation coefficient was calculated, and a one-way ANOVA test and a *t* test were conducted to determine the relationships between demographic characteristics and personal well-being.

For the section on decision-making patterns, we calculated the percentages of agreement and disagreement regarding each situation, and in conditions of disagreement, we calculated the percentages of final decisions that were made according to the participants’ own views versus those made in accord with the views of their significant others.

## 3. Results

### 3.1. Relationships between Personal Well-Being and Demographic Variables

Correlation tests were conducted, the results of which showed that neither age nor IQ level had a significant relationship with personal well-being (see Table 3). One-way ANOVA tests were used to examine the differences in the mean scores for personal well-being among the groups of different marital statuses, educational levels, and income. Personal well-being showed no significant difference from marital status [F(3, 166) = 0.776, *p* = 0.509], educational level [F(6, 163) = 2.029; *p* = 0.065], or income [F(5, 156) = 0.709; *p* = 0.617]. The results of the independent *t* tests also showed that neither gender nor living arrangements had a significant relationship with personal well-being.

### 3.2. Correlation of Self-Determination Competencies and Personal Well-Being

The possible scores for the self-determination competencies section ranged from 5 to 30. One participant scored 6 (the lowest of the 170 participants), whereas seven participants scored full marks (30), and the mean score was 20.2 (SD = 5.28). The possible scores on the Personal Well-Being Index ranged from 8 to 40. One participant scored 20 (the lowest of the participants’ scores), while 15 participants scored 40 (the maximum possible). The mean score was 32.96 (SD = 4.65).

A correlation test was conducted on the causal relationship between self-determination competencies (independent variable) and personal well-being (dependent variable). Table 3 presents the results, showing that self-determination competencies correlated significantly and positively with personal well-being (*r* = 0.313, *p* < 0.001).

### 3.3. Personal Goals

Among the participants, 138 (81%) reported their personal goals, with a total of 154 responses because some participants provided more than one goal. Again, we used Schalock and Verdugo’s QOL framework [22] to categorize the various types of personal goals. Investigator triangulation was carried out by the first author and the research assistant. We conducted a parallel data analysis. After a discussion and revisiting of the analysis, a consensus was ultimately reached. The results revealed that six QOL domains were covered. Two (1.3%) responses belonged to the QOL domain of emotional well-being; 60 (39%) belonged to the QOL domain of material well-being (39%); 18 (11.7%) belonged to the QOL domain of interpersonal relations; 40 (26%) belonged to the QOL domain of personal development; 29 (19%) belonged to the QOL domain of physical well-being; and 5 (3%) belonged to the QOL domain of social inclusion. Table 4 shows the types and frequencies of all of the personal goals.

### 3.4. Frequency of Agreement and Disagreement with Significant Others in Various Contexts

The total frequency of the decisions made by the 170 participants and their significant others in nine contexts was 1530, with 1519 responses (and 11 missing data items). Among the responses, 170 were reported as situations of disagreement (11.2% of the total number of responses). Table 5 presents the frequency of the participants’ agreement and disagreement with significant others in decision-making in the nine different contexts. The results demonstrate that situations of agreement in opinions were predominant, no matter what the context was. From among the various contexts, the three with the highest agreement levels were (1) choosing from the centre’s daily activities (95.3%), (2) making choices about further study (90.5%), and (3) choosing where to go out (89.9%). The contexts of choosing whether or whom to date (15.6%), of choosing a job (14.1%), and of choosing living arrangements (12.9%) had the three highest levels of disagreement. The results also demonstrated that in the situations of disagreement, the final decisions were made more frequently on the basis of the opinions of significant others than on the participants’ own views. However, overall, the results demonstrated that situations of disagreement were not predominant in the contexts of the most important decisions.

## 4. Discussion

The first hypothesis of this study—that the relationship between the self-determination competencies and the personal well-being of Chinese adults with ID was positively correlated—was supported. When controlling for age, IQ level, gender, marital status, living arrangements, educational level, and income, the results of this study suggest that a person’s self-determination competencies are positively correlated with his/her personal well-being, a discovery that echoes the results of studies conducted in other countries [24,25,26,27,28]. Those other studies demonstrated the merits of self-determination for the subjective well-being and personal satisfaction of people with ID, but none of them were undertaken with Chinese people with ID. The findings of this study, on the other hand, reflect the etic (universal) property of self-determination as an important QOL indicator in Chinese people with ID, thus providing important new support for the assumption that across various cultures self-determination is beneficial to people with ID. In China’s collectivist culture, self-determination competencies, although not used to advocating self-determination in right-based matters, are still important for individuals’ QOL [39]. Once individuals become more self-determined in terms of pursuing their personal goals, they tend to be more satisfied with life and feel happier. This speaks to the concerns of the intellectual disability service system in Hong Kong with regard to dimensional interventions to enhance self-determination in service users with ID.

Our findings revealed that the majority of participants possessed their own personal goals, and that their personal goals were diverse and spread across multiple dimensions. The second hypothesis was also supported. We know that goal pursuit is a multi-phase process that includes goal setting, planning, pursuing, adjusting, and achieving [20,40]. To be self-determined, a person has to learn to make adjustments when necessary at different stages in the process of pursuing goals. Unfortunately, to date, no systematic curriculum has been developed in Hong Kong, either in special education or in adult services, to assist people with ID in planning the pursuit of their goals. The literature shows that several empirical curricula and intervention packages for self-determination have been developed in Western countries, such as the Next S.T.E.P. [41], Whose Future Is It Anyway? [42], ChoiceMaker [43], TAKE CHARGE [44], and the Self-Determined Learning Model of Instruction (SDLMI) [45]. Most studies suggest that self-determination curricula are effective in enhancing the general self-determination skills of students with ID disabilities. However, the content of those existing programmes primarily emphasizes goal-setting and action-taking in a school context or at the point of transition to adulthood, whereas self-determination may actually be more important for adults making various major and minor decisions in different life situations. Because goal pursuit is a complex process that requires managing one’s personal and environmental situations and regulatory skills, there is a strong need for Hong Kong to develop a systematic programme/curriculum that is tailor-made to help adults enhance their self-determination. With regard to the training design, the content could cover a variety of skills learning such as goal-setting and planning skills, problem-solving skills, decision-making skills and self-regulatory skills. Hence, it is suggested that the elements of working memory, a main component of executive function, and emotion regulation be included in the instructional strategies as previous studies provided evidence that these two elements facilitate skills acquisition by people with ID [46,47]. Employing slogans as well as visual cues such as flow charts and videos during training would help people with ID acquire a better working memory as it would facilitate them to learn self-determined skills and apply those skills in their daily lives. It is also suggested that reappraisal strategies of emotion regulation be employed as they can increase the motivation of people with ID to solve problems and overcome the challenges encountered in pursuing goals. A supportive model of co-teaching [48] could be introduced in which disability personnel and parents collaborate to deliver training in order to maximize its effects.

Given that the autonomy support and well-being of people with ID is positively correlated [49], it is suggested that need-supportive practices by significant others (e.g., parents and paid carers) be introduced to help create a favourable interpersonal environment in which people with ID can feel more autonomous in exercising their self-determination. Need-supportive practices include showing understanding of the perspectives and situations of people with ID (e.g., showing empathy), showing appreciation, displaying patience, giving a rationale, providing emotional support, and offering information and strategies. One previous study found that satisfying parental intrinsic needs for competence, autonomy and relatedness could help those with ID adopt such need-supportive practices [50]. Similarly, another study suggested that in order to enhance teachers’ abilities to use a motivating style, their need satisfaction should first be fulfilled [51]. Hence, it was suggested that interventions be carried out to promote experiences of competence, autonomy and relatedness for parents and disability personnel. The reappraisal of emotion regulation strategy could be adopted in such interventions.

The third hypothesis—that the frequency of disagreements between adults with ID and their significant others would be higher when making major decisions—was not supported. The study’s results showed that, in a variety of contexts, and regardless of the levels of self-determination competencies that the participants possessed, their opinions were often consistent with those of their significant others. The results also indicated that the frequency of disagreement between people with ID and their significant others was very low. The assumptions related to culture may explain these findings. Some academicians claimed that people from collectivist cultures (such as Chinese people) internalized the demands of others who were close to them and were content to accept decisions made by others or to act on the demands of others [52]. If this is the case, the study’s participants may very often consider or internalize the thoughts and demands of their significant others when they make their choices in various contexts. Thus, their own opinions and those of their significant others would be in agreement.

In addition, according to Goode and Maloof [53], people in collectivist cultures value social interdependence rather than individual independence and take into consideration the interests of their families and other individuals when they make decisions. Chinese culture has a family-centred model of decision-making, and Chinese individuals may not feel disempowered under such a decision-making model [53]. This may explain why the participants who possessed a high level of self-determination competency usually reported having opinions consistent with those of their significant others and still achieving a high degree of personal well-being.

The results further indicated that the situations in which the participants and their significant others disagreed were not significantly related to major decisions. Among the nine contexts, those of dating, career choice, and living arrangements had the highest levels of disagreement between the two parties. These results do not seem to be in line with the findings of a previous study, which showed that the responses of the participants from all three parties—participants with ID, their parents, and disability personnel—indicated that the exercise of self-determination was conditional, depending on what kinds of decisions were made [32]. In contrast, this study found that the situations of disagreement in the contexts of major decisions were not more frequent than those of daily decisions. An assumption may explain this phenomenon. The process of decision-making was interactive and could have involved significant communication and discussion between the participants and their significant others. Then, when the participants were asked the questions, their answers may have depended on whether the participants perceived communication and compromise to be a process for reaching consistency or inconsistency. With the experience accumulated by this study’s findings, we therefore recommend that future studies explore the process and dynamics of decision-related communication in terms of the consistency/inconsistency that occurs at particular points of time in various situations. The dynamic interplay between people with ID and their significant others in the decision-making process increases the dimensions for intervention.

Our results showed that, in all of the contexts we studied, the issue of dating had the highest level of disagreement. This is in line with the findings of the studies conducted by Rushbrooke and colleagues [54] and Sullivan and colleagues [55], in which people with ID reported that they themselves generally did not control their intimate relationships, but instead their family members or paid carers did so, even though they themselves wanted to take charge over those relationships. Our participants with ID believed that one reason they did not have control over their relationships was that their significant others did not have confidence in their capacity to manage intimate relationships.

Finally, we should highlight the limitations of this study. The participants were recruited from disability service agencies, which meant that people with mild ID who were not using formal services might not have been contacted. Thus, the findings may not be generalizable to those who do not receive formal services. Another limitation was that the demographic data we collected did not include the socioeconomic background of the participants’ families, and family status may have an impact on the exercise of self-determination by people with ID.

## 5. Conclusions

In the field of international intellectual disability, the studies related to self-determination focus predominantly on educational contexts and the enhancement of children’s capacity for self-determination, and there is little emphasis on adult situations and on collectivistic cultures such as the Chinese culture. This study’s findings confirm a positive correlation between self-determination competencies and personal well-being, thus further supporting the notion that self-determination is beneficial to people with ID across various cultures. In addition, the study’s results contribute to the context of adulthood, including verifying the multidimensional aspect of the personal goals of adults with ID and gathering preliminary data on their (dis)agreement patterns for decision-making in different life situations.

## Figures and Tables

**Table 1:** Demographic Characteristics of Participants (N = 170).

Variables	Frequency	%
Gender (*n* = 170)		
Male	82	48.2
Female	88	51.8
Age group (*n* = 170)		
18–25	23	13.5
26–35	44	25.9
36–45	51	30
46–55	35	20.6
56–65	17	10
Types of disability other than ID (*n* = 44)		
Autism	11	6.5
Hearing disability	3	1.8
Mental illness	20	11.8
Physical disability	5	2.9
Visual disability	5	2.9
Marital status (*n* = 165)		
Single	152	89.4
Married	6	3.5
Divorced	6	3.5
Widowed	1	0.6
Living arrangement (*n* = 170)		
Living with family	79	46.5
Living in supervised hostel	83	48.8
Living alone	7	4.1
Living with friend(s)	1	0.6
Educational level (*n* = 166)		
Uneducated	3	1.8
Primary school	21	12.4
Secondary school	31	18.2
Special school	59	34.7
Vocational training centre	47	27.6
Other	5	2.9
Employment (*n* = 152)		
Integrated vocational training centre	7	4.1
Sheltered workshop	92	54.1
Integrated vocational and rehabilitation services	26	15.3
Supported employment	15	8.8
Open employment	12	7.1
Income (monthly, in HKD) (*n* = 162)		
≤1000	81	47.6
1001–2000	41	24.1
2001–3000	8	7.4
3001–4000	12	7.1
4001–5000	9	5.3
≥5001	11	6.5

**Table 2:** Decision-Making Patterns in Agreement/Disagreement Situations.

Responses to the Question ‘How Much Agreement Is There between Your Views and the Views of Your Significant Other in the Following Situations? When There Is Disagreement between You and Your Significant Other, Whose Opinion Do You Follow?’				
Situations	A	B	C	
	Agreement in Views (Please Go to Next Question)	Disagreement in Views (Please Go to ‘C’)	Opinion of Self (S)/ Opinion of Significant Other (O)	
Choosing from the centre’s daily activities				
Going out (e.g., where and when you go, and with whom)				
Daily use of money (e.g., what things to buy, and saving money)				
Further study (e.g., what you want to learn, and what course(s) you want to take)				
Career choice (e.g., what kind of job you want)				
Living arrangement (e.g., live in hostel or not, live with whom)				
Medical decisions (e.g., about dental treatment and surgery)				
Dating (e.g., dating or not, and who to date)				
Getting married and having children (if you have a boyfriend or girlfriend)				
		Total:	S:	O:

**Table 3:** Means, Standard Deviations, and Correlations of Self-Determination Competencies, Demographic Variables, and Personal Well-Being.

Variable	M	SD	1	2	3	4
1. Age	39.48	11.27	-	0.079	−0.101	−0.119
2. IQ level	60.53	7.67	0.079	-	0.068	−0.189
3. Self-Determination competencies	20.15	5.20	−0.101	0.068	-	0.313 **
4. Personal well-being	32.96	4.65	−0.119	−0.189	0.313 **	-

** *p* < 0.01.

**Table 4:** Types of Personal Goals.

QOL Domains (No. of Responses)	QOL Sub-Domains (No. of Responses)	Examples of Personal Goals
Emotional Well-being (2)	Happiness (2)	• Have a peaceful life
Material Well-being (60)	Career (22)	• Find a cashier job at bakery shop • Get a job in open job market • Find a job related to photography • Find a job related to logistics • Open a painting studio
	Finance (8)	• Save money
	Possession (24)	• By a mobile phone • Buy clothes
	Living Arrangement (6)	• Get a public housing unit • Live together with family
Interpersonal Relations (18)	Family (11)	• Visit family members in Canada
	Intimacy (3)	• Get married
	Interactions (4)	• Improve interpersonal relationship
Personal Development (40)	Self-ability (7)	• Learn to buy things independently • Take care of self
	Education and Skills (17)	• Learn to use a computer • Learn Putonghua • Learn more words
	Personal Competence (3)	• Perform solo dancing
	Sports Achievement (3)	• Be winner in a running contest
	Vocational Advancement (10)	• Get advancement in team work • Achieve good working performance
Physical Well-being (29)	Leisure and Hobbies (23)	• Travel around the world (first stop mainland China) • Travel overseas • Go to Disneyland
	Health-related (6)	• Keep healthy • Lose weight
Social Inclusion (5)	Community Roles (4)	• Be a volunteer
	Community Participation (1)	• Do charity work

**Table 5:** Frequency of Agreement and Disagreement about Decisions in Different Contexts.

Contexts	Agreement in Opinion		Disagreement in Opinion	
	Frequency	%	Frequency	%
Choosing from the centre’s daily activities (*n* = 170) Going out (*n* = 169) Daily use of money (*n* = 170) Doing further study (*n* = 169) Career choice (*n* = 170) Living arrangements (*n* = 170)	162 152 151 153 146 148	95.3 89.9 88.8 90.5 85.9 87.1	8 (S = 2; O = 6) 17 (S = 4; O = 13) 19 (S = 8; O = 11) 16 (S = 7; O = 9) 24 (S = 5; O = 19) 22 (S = 6; O = 16)	4.7 10.1 11.2 9.5 14.1 12.9
Medical decisions (*n* = 169) Dating (*n* = 167) Marriage and having children (*n* = 165)	150 141 146	88.8 84.4 88.5	19 (S = 4; O = 15) 26 (S = 9; O = 17) 19 (S = 7; O = 12)	11.2 15.6 11.5

Note. S = final decision made on the basis of the participant’s opinion. O = final decision made on the basis of the significant other’s opinion.

## Data Availability

The data presented in this study are available on request from the corresponding author.
